# Selective Electrocatalytic Production of Formic Acid from Plastic Waste Using a Nickel Metal–Organic Framework Constructed from a Biomass‐Derived Ligand

**DOI:** 10.1002/cssc.202402319

**Published:** 2024-12-12

**Authors:** Satarupa Das, Ting Zhang, Guy J. Clarkson, Marc Walker, Xufang Qian, Xia Long, Yixin Zhao, Richard I. Walton

**Affiliations:** ^1^ Department of Chemistry University of Warwick Gibbet Hill Road Coventry CV4 7AL UK; ^2^ School of Environmental Science and Engineering Frontiers Science Center for Transformative Molecules Shanghai Jiao Tong University Shanghai 200240 China; ^3^ Department of Physics University of Warwick Gibbet Hill Road Coventry CV4 7AL UK

**Keywords:** MOF, PET, Plastic upcycling, Electrocatalysis

## Abstract

A novel nickel‐based metal organic framework (MOF) [Ni(FDC)(CH_3_OH)_1.5_(H_2_O)_0.5_](H_2_O)_0.35_ (UOW‐6) utilizing biomass‐derived 2,5‐furan dicarboxylate (FDC) as a ligand is reported as an electrocatalyst for anodic ethylene glycol (EG) oxidation with cathodic hydrogen evolution. The MOF structure was analyzed using single crystal X‐ray‐diffraction, thermogravimetric analysis (TGA) and thermodiffractometry, to establish its structure and verify phase purity. The material was dropcast on carbon fiber paper as a catalyst, and by using a three‐electrode system, UOW‐6 requires only 1.47 V to attain a current density of 50 mA cm^−2^. During oxidation of the EG, UOW‐6 shows unprecedented selectivity towards formic acid with a Faradaic efficiency of 94 % and remarkable stability over 20 days. The combination of electrochemical measurements and *in situ* Raman confirmed *in situ* formed NiOOH at the surface of UOW‐6 as the catalytically active sites for EG oxidation. This work not only presents a pioneering application of FDC‐based MOFs for polyethylene terephthalate (PET) upcycling but also underscores the potential of electrocatalysis in advancing sustainable plastic valorization strategies.

## Graphical abstract

A metal‐organic framework constructed from the sustainably sourced ligand 2,5‐furandicarboxylate and nickel provides a highly active and selective catalyst from the oxidation of ethylene glycol: this is put to use in a flow cell with cathodic generation of hydrogen.

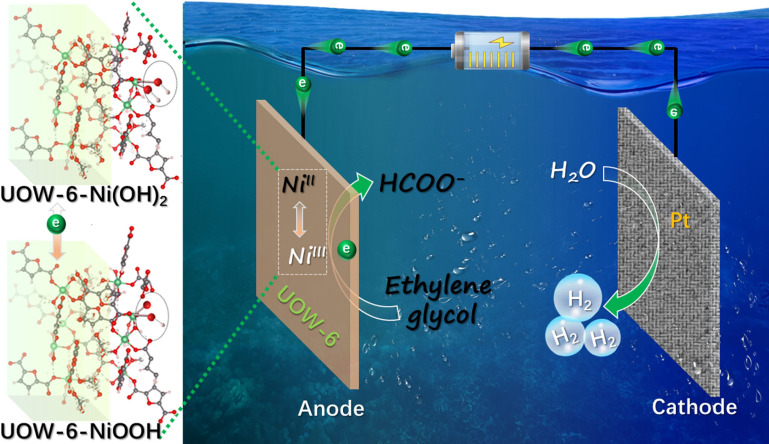

## Introduction

Water electrolysis, a cornerstone of renewable energy technologies, encompasses two pivotal reactions: the anodic oxygen evolution reaction (OER) and the cathodic hydrogen evolution reaction (HER).^[1]^ Molecular hydrogen (H_2_) stands out as a vital precursor in the chemical industry and a promising carbon‐free energy carrier. However, its predominant production from fossil fuels generates significant carbon dioxide emissions. While the OER appears ostensibly benign environmentally, its proton‐coupled‐electron‐transfer mechanisms inherently suffer from sluggish kinetics, exerting a dominant influence on the overall voltage of water electrolysis. Consequently, this impedes the electrolysis process, escalating energy consumption and requiring electrocatalysts that often use precious metals, altogether leading to hydrogen production costs.[^2^, ^3^] One way to overcome this limitation is to leverage the oxidation of small molecules with lower oxidation potential than water. Substituting the OER with alternative, more efficient anodic reactions present a tantalizing prospect for reducing overall energy expenditure.[^4^, ^5^, ^6^, ^7^, ^8^]

Plastic waste is generated and discarded on a huge scale annually, accumulating in landfills and aquatic ecosystems, and therefore presents a substantial threat to the environment and biodiversity.[^9^, ^10^, ^11^] Despite the global production of over 8 billion tons of plastics, less than 10 % presently undergoes recycling. Among these plastics, polyethylene terephthalate (PET) constitutes 13 % of the total output, acclaimed for its lightweight properties, chemical resistance, thermal stability, rendering it one of the most prevalent plastics worldwide.[^12^, ^13^] With escalating PET consumption, the proliferation of PET waste in the environment is inevitable, bringing uncertain long‐term repercussions. PET is produced through either the transesterification reaction of ethylene glycol (EG) with dimethyl terephthalate or the esterification reaction of EG with terephthalic acid (TPA).^[14]^ The available chemical recycling methods, such as hydrolysis, methanolysis, and glycolysis, offer promising solutions by breaking down PET into its original monomers for subsequent applications, aiming to achieve molecular‐level recovery,^[15]^ but these methods bring significant challenges, associated with high energy requirements, low efficiency, and complexity, limiting their practical application.[^15^, ^16^, ^17^] Hence, there is a significant demand for alternative sustainable methods for reforming plastics.

Recent studies have highlighted the potential of utilizing PET waste for generating valuable chemicals and hydrogen through solar driven processes.^[18]^ Similarly, electrocatalysis, powered by renewable energy sources, presents a promising avenue for efficient and selective alcohol oxidation. Transitioning from noble‐based to non‐noble transition metal‐based electrocatalysts holds significant promise for EG oxidation derived from PET waste. Not only are these materials abundant and cost‐effective, but they also exhibit broad applications and considerable prospects for development. In this context, electrocatalytic upcycling of PET plastic waste emerges as a promising avenue for replacing anodic OER to allow cathodic hydrogen production more efficiently.[^19^, ^20^, ^21^, ^22^, ^23^]

Recently, nickel has emerged as an excellent alternative to noble metals for electro‐oxidation reactions of small molecules in alkaline environments. Nickel compounds, including nitrides, phosphides, and chalcogenides, have often shown impressive electrocatalytic performance.^[24]^ Alongside this, there has been a notable effort aimed at exploring and harnessing biomass as an economical, renewable, and readily available feedstock or precursor for various chemical processes and applications.[^25^, ^26^] Within this context, lignocellulosic biomass stands out as a promising source from which a wide range of valuable chemicals can be derived. Among these compounds, 2,5‐furandicarboxylic acid (H_2_FDC) holds particular significance, as it can be synthesized through the selective oxidation of 5‐hydroxymethylfurfural (5‐HMF), a derivative of biomass.[^27^, ^28^] The incorporation of H_2_FDC, or its deprotonated form 2,5‐furandicarboxylate (FDC), as a ligand for the synthesis of metal‐organic frameworks (MOFs) presents a relatively unexplored avenue, compared to the large families of MOFs based on benzene‐derived linkers. The FDC ligand offers a sustainable and environmentally friendly alternative to the conventional nonrenewable ligands, such as benzene dicarboxylates (BDCs) derived from fossil fuels, typically employed in MOF construction.[^29^, ^30^] In this work we report the synthesis of a novel nickel‐based MOF with the FDC linker and demonstrate its use as a robust electrocatalyst for PET waste upcycling to formic acid. To the best of our knowledge, this is the first utilisation of a metal organic framework for this application. We show how the catalyst exhibits record high selectivity towards the C1 product formic acid with a Faradaic efficiency of 94 % along with stability of minimum 480 hours (20 days).

## Results and Discussion

**Figure 1:**
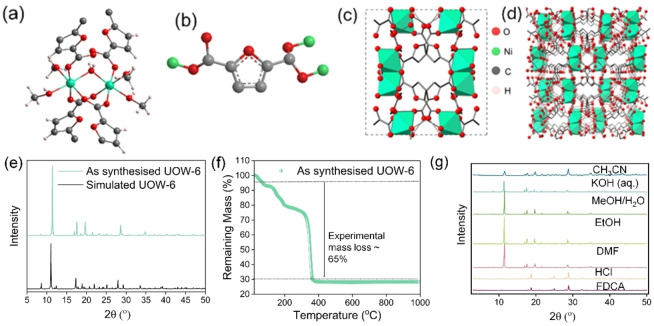
(a) The local environment of Ni ions in UOW‐6, (b) coordination mode of FDC linker, (c) connectivity of the dimers in the *bc* plane (d) 3D interconnected structure (e) The experimental and simulated XRD pattern of as synthesized UOW‐6, (f) TGA of UOW‐6 and (g) solvent stability test of UOW‐6 by powder XRD.

UOW‐6 (University of Warwick MOF structure number 6, following earlier rare‐earth MOFs we have reported elsewhere[^29^, ^31^]) is synthesized by a solvothermal reaction from a mixture of methanol and water using nickel nitrate hexahydrate as the metal source and 2,5‐furan dicarboxylic acid as the linker precursor. The MOF structure was determined by single crystal X‐ray diffraction, which reveals the material to have a chemical formula [Ni(FDC)(CH_3_OH)_1.5_(H_2_O)_0.5_](H_2_O)_0.35_. UOW‐6 has a monoclinic crystal system with a space group of *I*2/*a*. (Table S1) The structure, Figure 1a, contains a hexacoordinated nickel dimer which is bound via bridging water and two FDC ligands in which the Ni centres are octahedrally coordinated. One carboxylate group of the FDC binds via both oxygens, bridging the pair of Ni centres, which the other carboxylate binds via just one oxygen, with the free oxygen hydrogen bonding to a methanol that is also bound to Ni. The FDC binding mode is displayed in Figure 1b. The structure contains two methanol molecules bound to each nickel ion and unbound water (free water of crystallization). Overall the structure has three‐dimensional connectivity. (Figures 1c, d)

The MOF has two potential pore channels; however, the materials do not show any reasonable adsorption N_2_ isotherm which is likely because of the very narrow pore openings. In order to analyze this, pore visualization was carried out using CrystalExplorer software with an isovalue of 0.002 au following the method of Turner *et al*.^[32]^ As observed in Figure S1, the MOF does not have any visible porosity as the free space is blocked by very narrow restrictions. The powder XRD reveals good agreement with the simulated pattern from the single‐crystal structure determination, with no evident impurity peaks present, Figure 1e, while thermogravimetric analysis (TGA), Figure 1f, shows a set of mass losses that can be assigned as loss of solvent molecules, followed by combustion of the organic ligand to yield NiO (see Supporting Information). This demonstrates reasonable thermal stability, with complete structural decomposition not occurring until around 380 °C. The combustion of the organic components, which follows the loss of crystal water and bound methanol, results in a mass loss of 65 % which is in good agreement with the theoretical mass loss of 65.7 %. (Figure 1f) Thermodiffractometry using powder XRD was carried out and this shows a complete structural reordering as the solvent is lost in steps, but with retention of crystallinity (see Figure S2). Moreover, XRD of several batches of synthesized MOF shows high reproducibility. (Figure S3).

The stability of the MOF was investigated in a variety of solvents, Figure 1g, which showed stability in many solvents, including aqueous KOH, DMF, CH_3_CN, and MeOH/H_2_O which gives the possibility for diverse applications. Owing to its stable nature, the Ni‐FDC MOF was then tested for electrocatalytic ethylene glycol oxidation reaction in a three‐electrode set up under alkaline conditions. For comparison NiO and Ni(OH)_2_ were also studied as catalysts.

**Figure 2:**
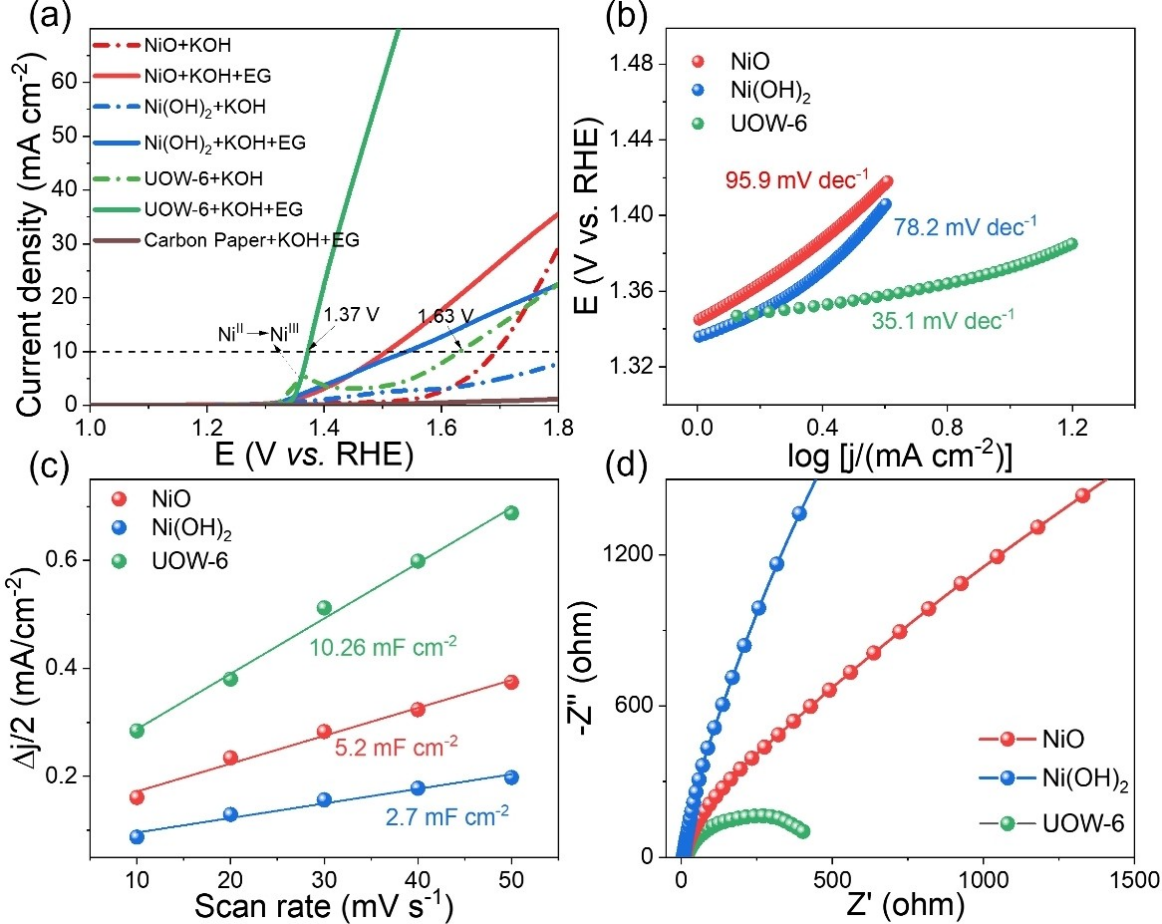
(a) Linear sweep voltammetry curves, (b) Tafel plots, (c) Capacitive current as a function of scan rates from 10 to 50 mV/s and (d) Nyquist plots for different Ni‐based catalysts.

In Figure 2a, the linear sweep voltammetry (LSV) curves demonstrate that all Ni‐based catalysts manifest relatively low onset oxidation potentials in EG hydrolysate, indicative of EG oxidation reactions for all. Notably, the UOW‐6 electrode exhibits the highest catalytic activity among the tested electrodes. Specifically, a current density of 10 mA cm^−2^ was attained at a potential of 1.37 V in EG hydrolysate, a value approximately 260 mV lower than that observed for the oxygen evolution reaction (OER). In the absence of EG, a discernible wave corresponding to the oxidation of Ni^II^ to Ni^III^ is solely observed with UOW‐6, consistent with the presence of Ni in its Ni^II^ oxidation state within the UOW‐6 structure.

Figure 2b illustrates that the UOW‐6 electrocatalyst displays a notably lower Tafel slope of 35.1 mV dec^−1^ and an onset potential of 1.34 V compared to other electrodes in EG hydrolysate, indicating its superior intrinsic electrocatalytic activity and rapid reaction rate for EG oxidation. A comparison with previously reported non‐noble metal phosphide and oxide electrocatalysts for ethylene glycol oxidation, is presented in Table S2, which further underscores the competitive catalytic performance of the UOW‐6 electrocatalyst under analogous conditions. As observed in the previous report by Huang and coworkers, a bifunctional electrode of Ni_3_N/W_5_N_4_ shows an outstanding stability of ~300 h, however it suffers from comparative low Faradaic efficiency and a higher onset potential.^[22]^ Moreover, the fabrication of the catalyst requires multiple steps and the possible issue of reproducibility of such a composite must also be considered. In another report by Zhou *et al*., nickel cobalt phosphide grown on to a nickel foil shows remarkable current density, however, the catalyst shows stability up to only 75 h,^[33]^ whereas in our case the MOF catalyst retains stability over 480 h, which to our knowledge is higher than in any other previously reported catalysts for this application.

Based on the cyclic voltammetry at different scan rates, the UOW‐6 electrode exhibits a higher double‐layer capacitance (*C*_dl_) of 10.26 mF cm^−2^, as depicted in Figure 2c, implying that it possesses abundant electrochemical catalytic active sites for EG oxidation reaction. The lower charge transfer resistance and larger electrochemical active surface area of UOW‐6 electrocatalyst compared to that of other Ni‐based electrodes endow it with excellent catalytic properties for ethylene glycol oxidation. This assertion is corroborated by the Nyquist plots presented in Figure 2d, wherein the UOW‐6 electrocatalyst demonstrates the lowest transfer resistance, consistent with its superior catalytic activity among the tested Ni‐based electrodes. Considering these findings, we focus the following studies of the catalytic activity for ethylene glycol oxidation on the UOW‐6 electrocatalyst.

**Figure 3:**
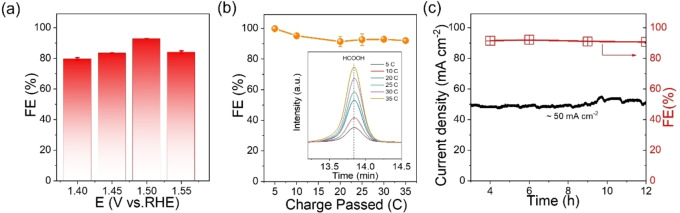
(a) Faradaic efficiency of formate production at the different electrolysis potentials; (b) Corresponding Faradaic efficiencies at the different amounts of charge passed; (c) *i*‐*t* curve and Faradaic efficiency in a long‐term stability test of UOW‐6 electrocatalyst in an H‐type cell.

Considering the large current densities, we selected UOW‐6 for the oxidation of ethylene glycol, where it can achieve more than 80 % selectivity towards formate under different voltage (1.40, 1.45, 1.50, and 1.55 V vs. RHE) conditions. At a voltage of 1.5 V vs RHE, the Faradaic efficiency (FE) for formate reaches its peak at approximately 93 %, as depicted in Figure 3a.

With the applied voltage fixed at 1.5 V vs RHE, Figure 3b shows the FE with different charges passed alongside the corresponding HPLC chromatograms. The FE for formate remains consistently above 90 %, indicating that the as‐prepared UOW‐6 electrode possesses an excellent selectivity and stability. Another pivotal metric for evaluating the efficacy of electrocatalysis is long‐term stability. Over the course of a 12‐hour test conducted in an H‐type cell, the UOW‐6 electrode exhibits continuous operation without any discernible degradation in activity or selectivity, as evidenced by Figure 3c. In order to assess the integrity of the catalyst, several control tests were performed, and X‐ray photoelectron spectroscopy was measured. The MOF catalyst was soaked into a series of solutions for 24 hrs – 1 M KOH, 1 M KOH+0.5 M H_2_O_2_ and 1 M KOH+0.1 M EG+0.5 M H_2_O_2_ and compared against pristine UOW‐6. As observed from the XPS spectra, in all cases the catalyst retains the same Ni oxidation state as evident from Figure S4, and Tables S3–7. In addition, inductively coupled plasma optical emission spectrometry (ICP‐OES) shows negligible leaching (0.4 ppm) of the nickel into the electrolyte, further proving the chemical stability of the MOF.

**Figure 4:**
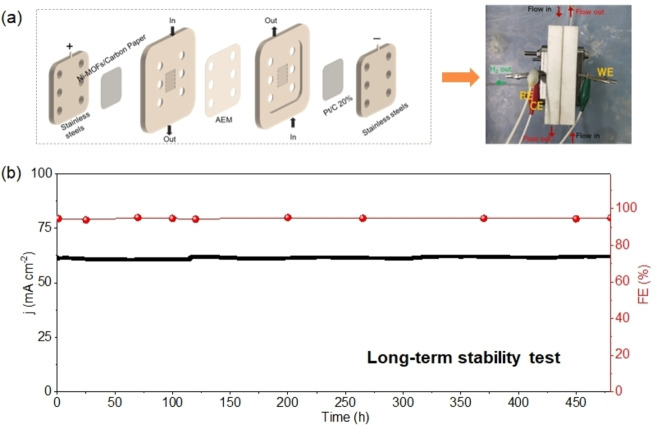
(a) Illustration of each component principle of the self‐made flow reaction device and digital image of it; (b) *i–t* curve and Faradaic efficiency in long‐term stability test of UOW‐6 electrocatalyst in flow reaction device.

In order to provide additional evidence of its electrochemical stability, we subjected the UOW‐6 electrode to testing using a custom‐designed flow reaction device, as depicted in Figure 4a. This device offers the advantage of maintaining a constant renewal of the electrolyte, thereby mitigating the accumulation of reaction products on the electrode surface that could adversely influence electrocatalytic performance.

**Figure 5:**
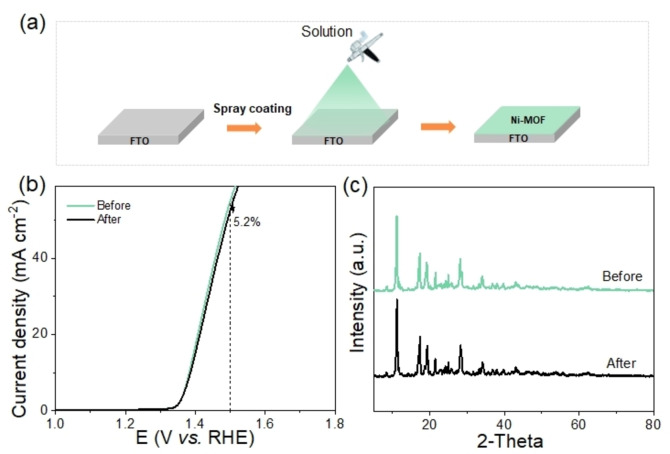
(a) Schematic diagram of spray UOW‐6 catalyst on FTO surface; (b) LSV curves and (c) XRD pattern of UOW‐6 before and after electrocatalytic reaction.

Under these conditions, a stable current density of approximately 61.0 mA cm^−2^ was attained at a cell voltage of 1.7 V (without *iR* correction). Remarkably, the UOW‐6 electrode exhibits outstanding stability over an extended period, as demonstrated by a long‐term test lasting 480 hours (Figure 4b, Table S2), during which it maintains a FE of approximately 94 % surpassing many other well‐known reported catalysts. The electrocatalytic stability of the UOW‐6 has been successfully demonstrated; however, the structural stability remains unexplored. To investigate this aspect, we employed a method wherein UOW‐6 ink was spray coated onto the surface of fluorine‐doped tin oxide (FTO) for electrochemical reaction, as illustrated in Figure 5a.

After prolonged reaction periods, slight decreases in the LSV results were observed, indicating potential alterations in the catalytic performance, Figure 5b. To assess the structural stability of the UOW‐6 catalyst post‐reaction, powder X‐ray diffraction (XRD) analysis was performed on the catalyst after removal from the electrode. This reveals the retention of crystallinity and structure of the UOW‐6, Figure 5c, showing that while minor alterations in electrocatalytic performance may occur, the overall structural integrity of the UOW‐6 catalyst remains intact. Transmission electron microscopy was also used to confirm the stability of the material after electrocatalysis, which shows that the characteristic needle‐like morphology and crystallinity was retained (Figure S5).

**Figure 6:**
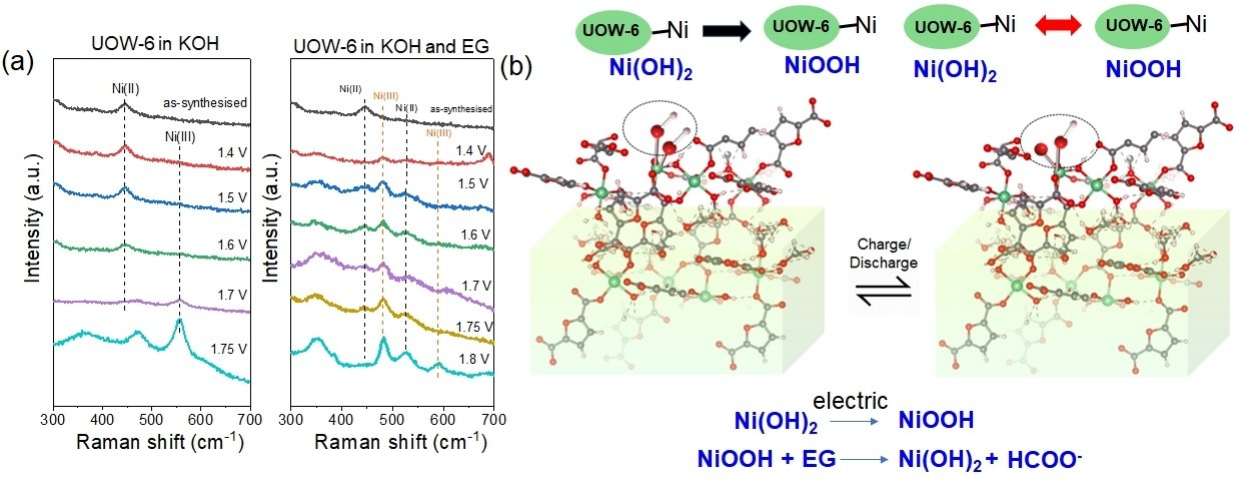
(a) *In situ* Raman spectra collected under chronoamperometry test for two different reaction conditions using UOW‐6 as the catalyst. (b) Plausible mechanism for EGOR at the UOW‐6 surface.

The selectivity of the electrocatalyst towards forming solely formate as the oxidation product was confirmed using solution NMR spectroscopy (Figure S6). In order to further probe the catalytic mechanism, *in situ* potential dependent Raman spectroscopy was conducted in two different conditions: one in the presence of just 1 M KOH (without ethylene glycol) and one with both 1 M KOH and 0.1 M ethylene glycol (Figure 6a). At a potential below 1.7 V, a Raman peak appears at approximately 447 cm^−1^, corresponding to the A_1g_ stretch of Ni‐OH. In the case of the oxygen evolution reaction (OER), when the potential is increased, two new peaks emerge at 470 and 557 cm^−1^. These peaks are associated with the E_g_ bending and A_1g_ stretching vibrations, respectively, of Ni^III^‐O in NiOOH.[^34^, ^35^, ^36^, ^37^, ^38^] The Raman peak intensity of Ni(OH)₂ is significantly weaker compared to that of NiOOH.

In the case of the ethylene glycol oxidation reaction (EGOR), when the applied potential exceeds 1.4 V, a distinct E_g_ bending around 472 cm^−1^, indicative of the presence of NiOOH, is observed (Figure 6b). In addition, the features of Ni^II^‐OH stretching and bending modes reappear which essentially means rapid consumption of NiOOH in the presence of ethylene glycol. This observation indicates that in the OER, the applied anodic potential keeps the nickel in an oxidized state, favoring the formation and maintenance of NiOOH over Ni(OH)_2_. The applied bias serves primarily to regenerate NiOOH, which acts as a chemical oxidant. Ni(OH)_2_ undergoes oxidation to form NiOOH, which then abstracts an α‐hydrogen atom from the adsorbed alcohol. Following this, NiOOH is converted back to Ni(OH)_2_. Similar observations have been identified by Kang *et al*. who studied a NiCu/nickel foam catalyst.^[39]^

We finally studied the electrocatalysis at a range of cell voltages to further understand its performance. At potentials up to 1.8 V the Faradaic efficiency of formate production at the anode was maintained at ~93–95 %, and only dropped to 85–90 % at higher potentials of 1.9 V and 2.0 V (Figure S7). The drop in efficiency at higher applied voltages is likely due to further oxidation of the products or oxidation of water, as has previously been proposed in other systems.[^20^, ^40^]

## Conclusions

We have synthesized a biomass linker‐derived MOF by a mild solvothermal reaction, that has a unique topology. The material has successfully been used for effective electrocatalytic upcycling of ethylene glycol derived from PET waste through anodic ethylene glycol oxidation and concurrent cathodic hydrogen production. The MOF catalyst, without any treatment following its synthesis, showcases an impressive selectivity and Faradaic efficiency of 94 % towards formic acid production. *In situ* Raman spectroscopy reveals that NiOOH at the surface UOW‐6 is the likely active site for EG electrocatalytic oxidation. The catalyst shows a remarkable stability of a minimum of 20 days under the bias in alkaline electrolyte. This study paves the way for the development of practical solutions for waste valorization and sustainable chemical synthesis.

CCDC 2367200 contains the supplementary crystallographic data for this paper, available at https://www.ccdc.cam.ac.uk/. All other data will be made available upon request from the authors.

## Conflict of Interests

The authors declare no conflict of interest.

1

## Supporting information

As a service to our authors and readers, this journal provides supporting information supplied by the authors. Such materials are peer reviewed and may be re‐organized for online delivery, but are not copy‐edited or typeset. Technical support issues arising from supporting information (other than missing files) should be addressed to the authors.

Supporting Information

## Data Availability

CCDC 2367200 contains the supplementary crystallographic data for this paper, available at https://www.ccdc.cam.ac.uk/. All other data will be made available upon request from the authors.
