# Supplementary material for: Selective Electrocatalytic Production of Formic Acid from Plastic Waste Using a Nickel Metal–Organic Framework Constructed from a Biomass‐Derived Ligand
Source: ChemSusChem. 2024 Dec 12;18(8):e202402319. doi: 10.1002/cssc.202402319 (PMC11997925; doi:10.1002/cssc.202402319)
Supplement: Supplementary file 1 — Supporting Information [file CSSC-18-e202402319-s001.pdf]

ChemSusChem

Supporting Information

Selective Electrocatalytic Production of Formic Acid from Plastic Waste Using a Nickel Metal–Organic Framework Constructed from a Biomass-Derived Ligand

Satarupa Das, Ting Zhang, Guy J. Clarkson, Marc Walker, Xufang Qian, Xia Long, Yixin Zhao,* and Richard I. Walton*

Selective Electrocatalytic Production of Formic Acid from Plastic Waste Using a Nickel Metal–Organic Framework Constructed from a Biomass-Derived Ligand

Satarupa Das,[a§] Ting Zhang,[b§] Guy. J. Clarkson,[a] Marc Walker,[c] Xufang Qian,[b] Xia Long,[b] Yixin Zhao[b,*] and Richard I. Walton[a,*]

[a] Department of Chemistry, University of Warwick, Gibbet Hill Road, Coventry CV4 7AL, UK
E-mail: r.i.walton@warwick.ac.uk

[b] School of Environmental Science and Engineering, Frontiers Science Center for Transformative Molecules, Shanghai Jiao Tong University, Shanghai 200240, China
E-mail: yixin.zhao@sjtu.edu.cn

[c] Department of Physics, University of Warwick, Gibbet Hill Road, Coventry CV4 7AL, UK

§ These authors contributed equally

Table of Contents

Experimental Section..**S3**

Synthesis of Ni-FDC MOF..**S3**

Materials characterization..**S3**

Electrochemical evaluation..**S4**

In situ Raman spectroscopy..**S5**

Supplementary Figures..**S6**

Supplementary Tables..**S11**

References..**S16**

EXPERIMENTAL SECTION

Chemicals

Nickel nitrate hexahydrate (Sigma Aldrich, 99.99%), 2,5-furan dicarboxylic acid (Alfa Aesar, 98%), sodium hydroxide (Fischer Scientific, 98%), potassium hydroxide (Sinopharm Group Co., Ltd. 99%), ethylene glycol (Sigma Aldrich, >99%, GC), hydrogen peroxide, methanol, de-ionized water was used as supplied.

Synthesis of Ni-FDC MOF (UOW-6)

60 mg of nickel nitrate hexahydrate, 87 mg of furan 2,5-dicarboxylic acid and 25 mg of sodium hydroxide Were placed together in a ~10 mL Teflon container and then, 2 mL of methanol was added. The resultant mixture was stirred for 5 minutes. 1 mL of de-ionized water was added and stirred for another 5 minutes. The Teflon container was transferred to a solvothermal autoclave and reaction was carried with a ramping rate of 5 ˚C/min to 110 ˚C where it was held 48 hours. The reaction was cooled at a ramping rate of 3 ˚C/min before the solid product was recovered by suction filtration and washed with deionized water and methanol and allowed to dry in air. Powder XRD of several batches of the synthesized MOF shows highly reproducibility. (Figure S3)

Materials characterization

Single crystal XRD: A suitable crystal was selected and mounted on a Mitegen head with Fomblin oil and placed on a Rigaku Oxford Diffraction Synergy-S diffractometer with a dual source equipped with a Hybrid pixel array detector. The crystal was kept at 100(2) K during data collection. Using Olex2 [1] the structure was solved with the ShelXT structure solution program using intrinsic phasing and refined with the ShelXL refinement package using Least Squares minimization. [2] Crystal void visualisation was carried out using CrystalExplorer software. [3] The void space was mapped using an isovalue of 0.002 au.

X-ray powder diffraction measurements were conducted with a 3rd generation Malvern Panalytical Empyrean instrument featuring multicore (iCore/dCore) optics and a Pixcel3D detector operating in 1D receiving slit mode. The source of radiation was a Cu tube emitting Cu $K_{\alpha 1/2}$ rays with a wavelength of 1.5418Å. Data collection spanned from 2 theta angles of 3° to 60°, with a step size of 0.0131° and a counting time of 2.5 seconds per step. Variable temperature XRD was conducted using Bruker D8 Advance diffractometer. Thermogravimetric (TGA) analysis was conducted using a Mettler Toledo TGA/DSC1 instrument under ambient air pressure with a heating rate of 10 °C per minute. The samples underwent heating in air from 25 to 1000 °C. N_2 adsorption

isotherms were measured at 77 K using a Micromeritics ASAP2020 apparatus, with the sample pretreated under vacuum at 120 °C for 3 h to remove water. The BET method was utilized to calculate the apparent surface area. X-ray photoelectron spectroscopy (XPS) was measured using a Kratos Axis Ultra DLD spectrometer which possesses a base pressure below 1 x 10^{-10} mbar. The samples were attached to electrically conductive carbon tape, mounted on to a sample bar with a layer of filter paper between the samples and the sample bar to ensure electrical isolation The XPS measurements were performed with the sample being illuminated using a monochromated Al Kα X-ray source ($h\nu$ = 1486.7 eV) at room temperature with a take-off angle of 90° with respect to the surface parallel. The core level spectra were recorded using a pass energy of 20 eV (resolution approx. 0.4 eV), from an analysis area of 300 × 700 μm^2. The work function and binding energy scale of the spectrometer were calibrated using the Fermi edge and $3d_{5/2}$ peak recorded from a polycrystalline Ag sample prior to the commencement of the experiments. To prevent surface charging the surface was flooded with a beam of low energy electrons throughout the experiment and this necessitated recalibration of the binding energy scale. To achieve this, the C-C/C-H component of the C 1*s* spectrum was referenced to 285.0 eV. The spectra were analysed in the CasaXPS package using Shirley backgrounds and mixed Gaussian-Lorentzian (Voigt) lineshapes. For compositional analysis, the analyser transmission function was measured using clean metallic foils to determine the detection efficiency across the full binding energy range.

NMR spectra were collected on a Bruker Ascend 400MHz, with the test sample was dissolved in D_2O.

TEM images were collected using a Thermo Fisher Scientific (FEI) Talos 200S microscope. The test sample was first dispersed in ethanol and then drip-coated onto a carbon film-supported copper grid.

Electrochemical evaluation.

All electrochemical experiments were performed on an electrochemical workstation (CHI 660, CH Instruments, Inc.). The 20% Pt/C (Alfa Aesar) powder catalyst was dispersed in an aqueous isopropanol solution (50%) with Nafion@ solution by sonification and sprayed on carbon fiber paper (CFP, HCP120, 0.21 $\pm$ 0.01nm) with a mass loading of 1 mg cm^{-2}. The geometric area of UOW-6 electrode and CFP-supported 20% Pt/C is 2 cm × 2 cm for all experiments. Ag/AgCl was selected as a reference electrode in a three-electrode system. All potentials measured against Ag/AgCl were converted to the reversible hydrogen electrode (RHE), using $E_{RHE}= E_{Ag/AgCl} + 0.197 +0.0592pH$. The EG oxidation process was carried out at potentiostatic mode without

iR compensation. The amount of formic acid product was detected by HPLC. The Faradaic efficiency (FE) for EG oxidation was calculated using Eq. (1) (n is the number of electron transfers for each value-added chemical formation).

$$FE(\%) = \frac{n \times product(mol) \times 96485\ (C/mol)}{Total\ charge\ passed\ (C)} \times 100\% \quad \text{.......................... Equation 1}$$

***In situ* Raman spectroscopy.**

In situ electrochemical Raman spectroscopy was measured via a confocal microscope (Invia Reflex, Renishaw, UK) combined with an electrochemical workstation (CHI 660, CH Instruments, Inc.). The excitation laser wavelength is 532 nm, the laser power was 50% 1 mW, the exposure time was 10s, with 3 accumulations, and a 10X objective was used to detect and collect the scattering signal. All Raman spectra were obtained within a spectra-electrochemical quartz cell. The calibration was conducted with a silicon wafer at 520 cm^{-1}. UOW-6 ink was spray coated onto the surface of fluorine-doped tin oxide (FTO), Pt wire and Ag/AgCl was used as working electrode, counter electrode and reference electrode, respectively, in a three-electrode system.

Supplementary Figures.

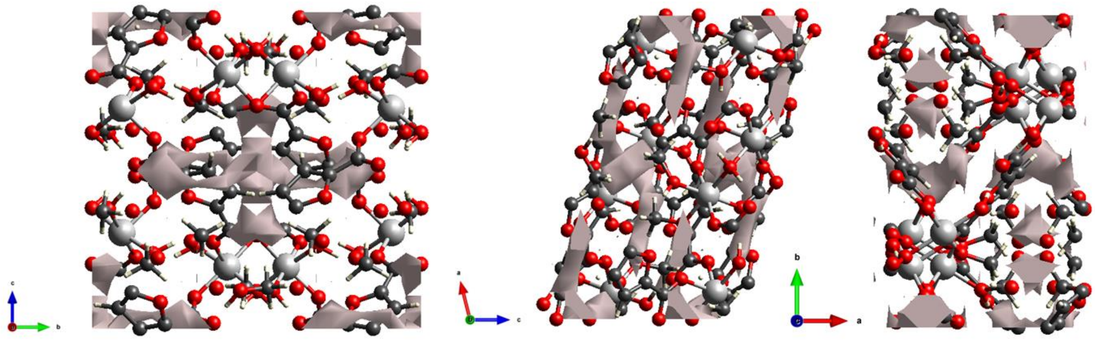

Figure S1. Visualisation of pore channels of UOW-6 along *a*, *b*, and *c* directions, respectively. The void surfaces were mapped using an iso value of 0.002 au (corresponding approximately to a smoothed van der Waals surface). This calculation provides a realistic upper limit to the actual space accessible to the guest molecules in a potential porous system.1 au of electron density = 6.748 e Å^{-3}.

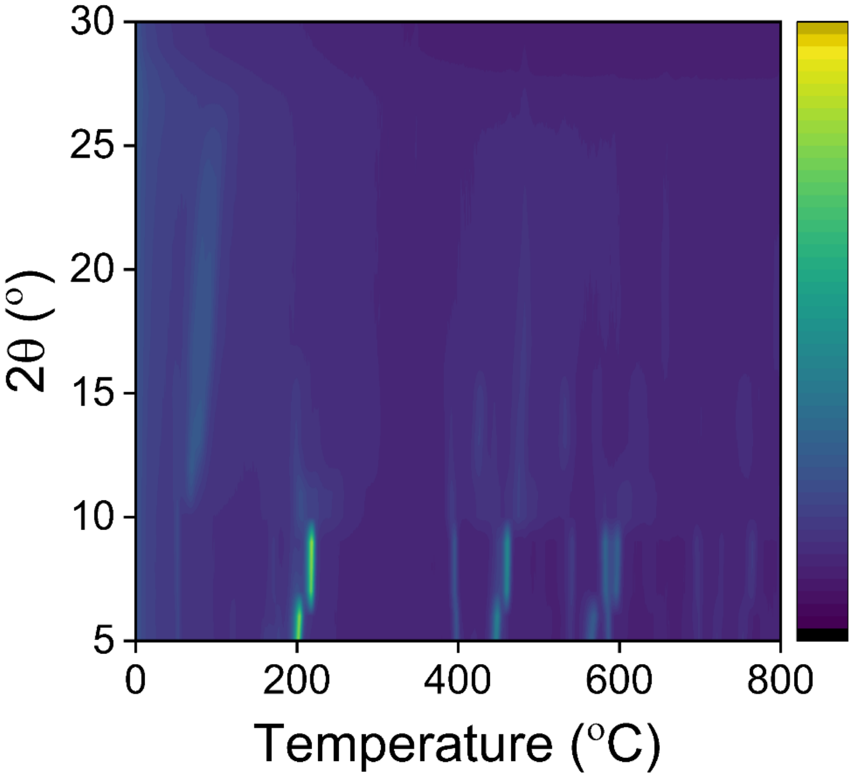

Figure S2. Thermodiffractometry heatmap of UOW-6 (Cu K$\alpha_{1/2}$ radiation)

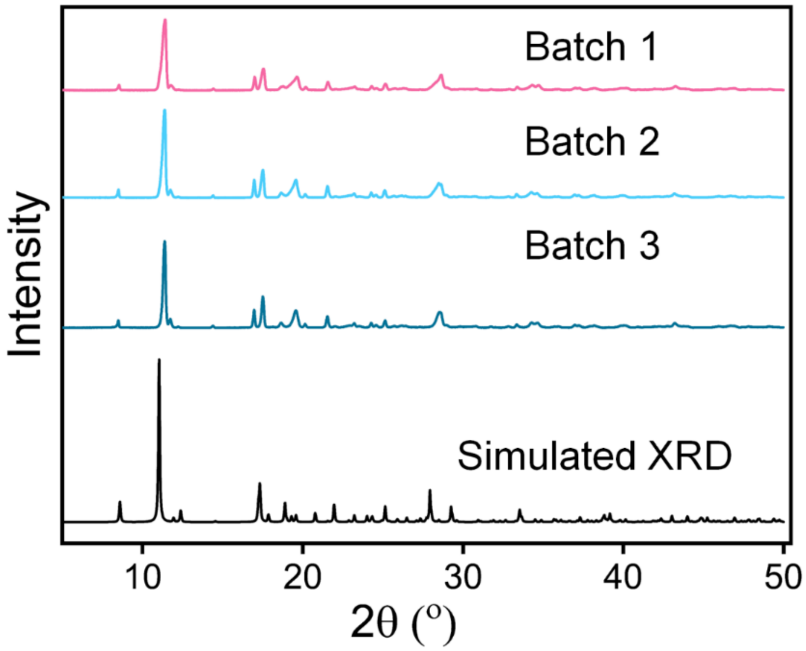

Figure S3. Reproducibility test of synthesis of UOW-6.

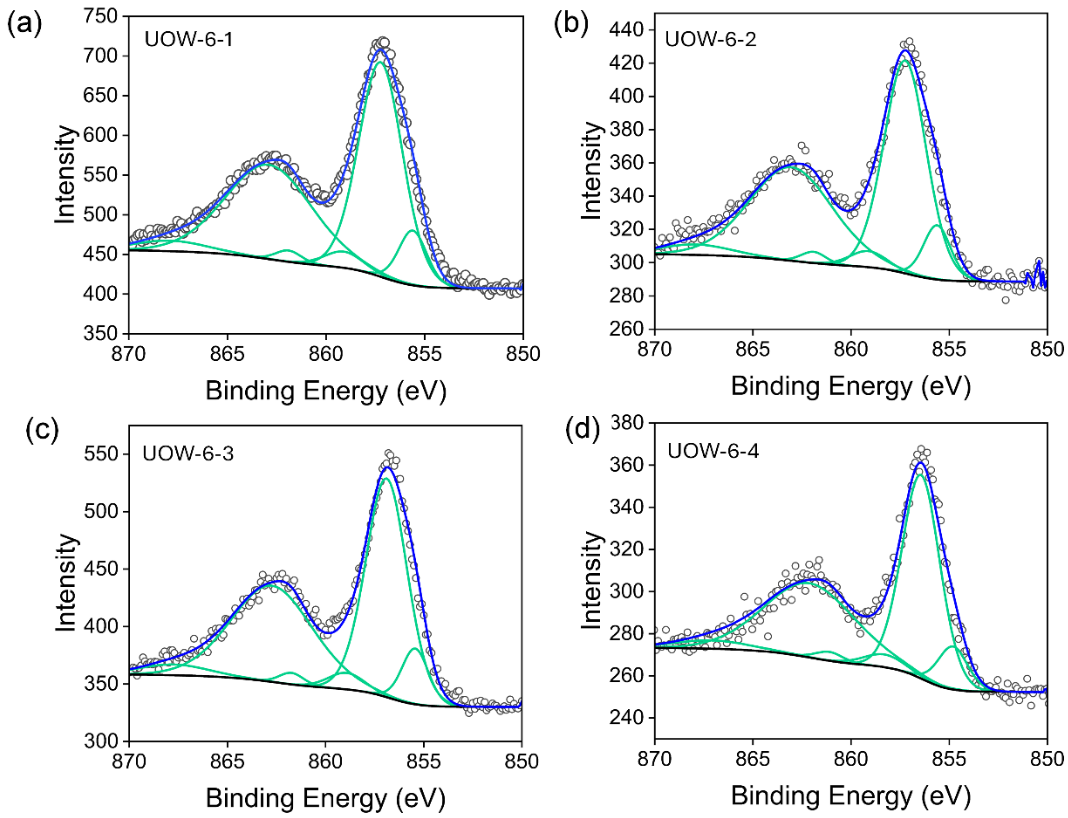

Figure S4. XPS measurements for UOW-6 in different media. (Details in Table S3-S7

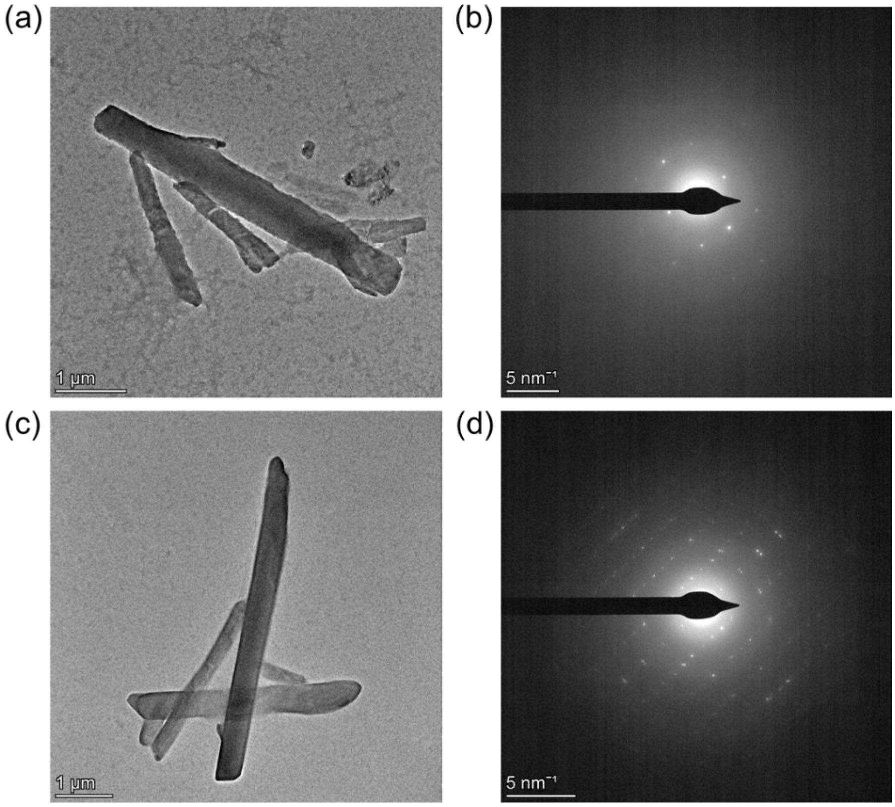

Figure S5: TEM images and corresponding SAED patterns of UOW-6 (a, b) before and (c, d) after electrocatalysis.

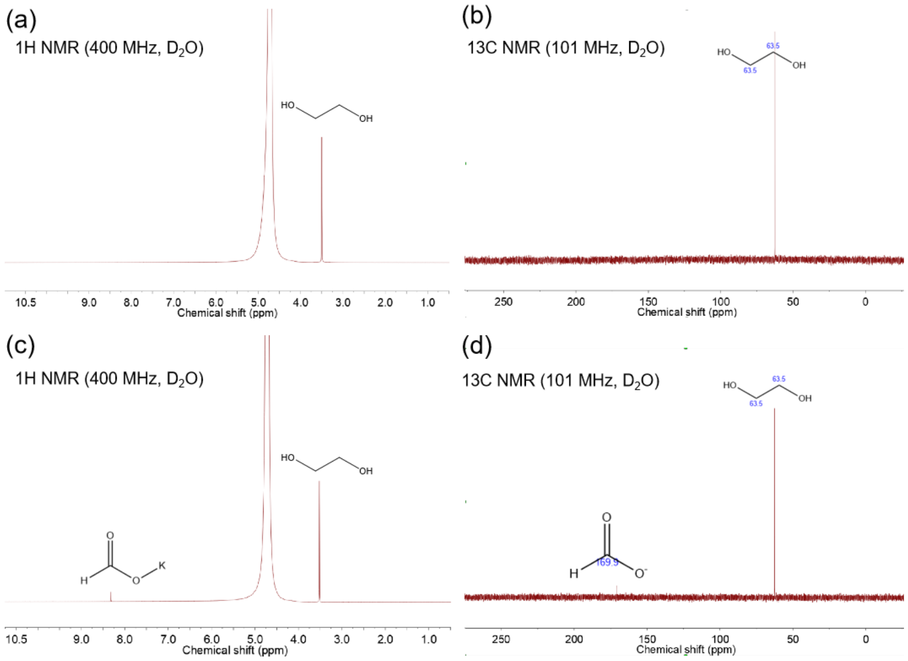

Figure S6: NMR spectra of electrolyte (a, b) before and (c, d) after electrocatalysis.

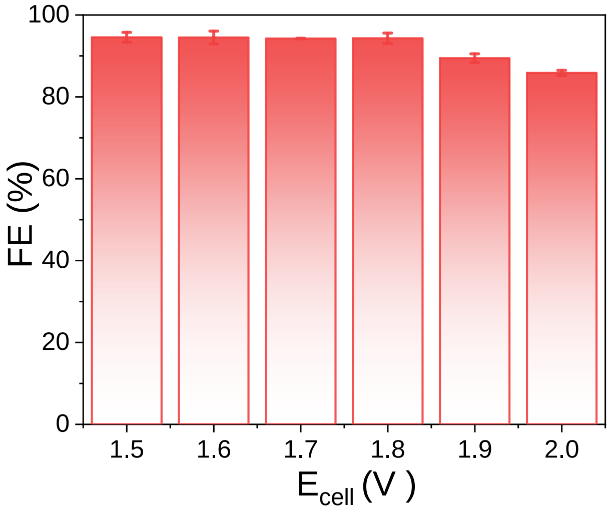

Figure S7. Faradaic efficiency of formate production for the flow reaction device at the different cell potentials

Table S1. Crystal data and structure refinement data of UOW-6

**Structure Code**	**UOW-6**
**Empirical formula**	$C_{7.5}H_9NiO_{7.85}$
**Formula weight /gmol^{-1}**	283.46
**Radiation**	MoKα (λ = 0.71073)
**Temperature**	100(2) K
**Crystal system**	monoclinic
**Space group**	*I*2/*a*
**Crystal density / gcm^{-3}**	1.785
a **/Å**	9.9768(3)
b **/Å**	14.2781(3)
c **/Å**	15.2707(4)
α **/°**	90
β **/°**	104.083(3)
γ **/°**	90
**Volume /Å^3**	2109.92(10)
**Z**	8
**2θ range for data collection/°**	5.086 to 66.928
**Absorption coefficient**	1.864 mm^{-1}
**Max. and min. transmission**	0.635 and 0.368
**Data/restraints/parameters**	3731/3/169
**Absorption correction**	spherical harmonics - frame scaling
***F*(000)**	1158.0

**Crystal size /mm^3**	0.2 × 0.1 × 0.03 green block
**Index ranges**	-14 ≤ h ≤ 15, -21 ≤ k ≤ 21, -22 ≤ l ≤ 21
**Reflections collected**	20548
**Goodness-of-fit on F^2**	1.046
**Independent reflections**	3731 [R_{int} = 0.0262, R_{sigma} = 0.0206]
**Final R indexes [I>=2σ (I)]**	R_1 = 0.0282, wR_2 = 0.0697
**Final R indexes [all data]**	R_1 = 0.0344, wR_2 = 0.0719
**Largest diff. peak/hole / e Å^{-3}**	0.57/−0.49

Table S2. Comparation of the electrocatalytic performance of non-noble electrocatalysts for ethylene glycol oxidation under alkaline conditions.

Electrocatalyst	Supporting electrolyte	Current density (mA cm^{-2})	Voltage (V_{RHE})	Faradaic efficiency (%)	Stability (h)	Ref.
**$NiCo_2O_4$**	1 M KOH	50	1.45	90	12	4
**$CuCo_2O_4$**	1 M KOH	100	1.50	93	20	5
**$CoNi_{0.25}P$**	1 M KOH	350	1.70	91.7	75	6
**Co−Ni_3N**	1 M KOH	10	1.18	92	15	7
**Ni_3N/W_5N_4**	1 M KOH	120	1.60	85	300	8
**CuO**	1 M KOH	10	1.38	85	-	9
**$CoSe_2$/NF**	1 M KOH	/	/	97	30	10
**OMS Ni_1−CoP**	1 M KOH	10	1.52	96	10	11
**Cu(F)@CuO@$Ni(OH)_2$**	1 M KOH	50	1.36	/	20	12
**NiSe**	1 M KOH	50	~1.4	80	48	13
**NiCo-SS-et**	1 M KOH	50	1.68	>80	10	14
**UOW-6**	1 M KOH	50	1.47	94	480	This work

Table S3. XPS analysis parameters for UOW-6 in different conditions and corresponding surface elemental compositions

		Atomic Percentage of elements			
**Sample**	**Experiment Label**	**Ni**	**C**	**O**	**K**
Nickel MOF	UOW-6-1	7.2	43	32.5	17.3
Nickel MOF+ 1 M KOH	UOW-6-2	3.9	39.3	32.2	24.6
Nickel MOF+1M KOH+ 0.5 M H_2O_2	UOW-6-3	4.6	43.4	30.5	21.4
Nickel MOF+1M KOH+ 0.1 M EG+ 0.5M H_2O_2	UOW-6-4	3.1	38.8	39.4	18.7

Tables S4-S7. XPS binding energies for UOW-6 after exposure to different conditions (see Table S3)

UOW-6-1		
Binding energy (eV)	% of region	Bonding environment
855.6	7.4	Ni 2p3/2 - $Ni(OH)_2$
857.23	45.2	Ni 2p3/2 - $Ni(OH)_2$
859.1	3	Ni 2p3/2 - $Ni(OH)_2$
861.9	1.4	Ni 2p3/2 - $Ni(OH)_2$
862.9	39.3	Ni 2p3/2 - $Ni(OH)_2$
867.9	3.7	Ni 2p3/2 - $Ni(OH)_2$

UOW-6-2		
Binding energy (eV)	% of region	Bonding environment
855.62	7.4	Ni 2p3/2 - $Ni(OH)_2$
857.24	45.2	Ni 2p3/2 - $Ni(OH)_2$
859.11	3	Ni 2p3/2 - $Ni(OH)_2$
861.91	1.4	Ni 2p3/2 - $Ni(OH)_2$
863.05	39.3	Ni 2p3/2 - $Ni(OH)_2$

867.91	3.7	Ni 2p3/2 - Ni$(OH)_2$

UOW-6-3

Binding energy (eV)	% of region	Bonding environment
855.44	7.4	Ni 2p3/2 - Ni$(OH)_2$
	45.2	Ni 2p3/2 - Ni$(OH)_2$
858.93	3	Ni 2p3/2 - Ni$(OH)_2$
861.73	1.4	Ni 2p3/2 - Ni$(OH)_2$
862.73	39.3	Ni 2p3/2 - Ni$(OH)_2$
867.73	3.7	Ni 2p3/2 - Ni$(OH)_2$

UOW-6-1

Binding energy (eV)	% of region	Bonding environment
854.81	7.4	Ni 2p3/2 - Ni$(OH)_2$
856.44	45.2	Ni 2p3/2 - Ni$(OH)_2$
858.31	3	Ni 2p3/2 - Ni$(OH)_2$
861.11	1.4	Ni 2p3/2 - Ni$(OH)_2$
862.11	39.3	Ni 2p3/2 - Ni$(OH)_2$
867.11	3.7	Ni 2p3/2 - Ni$(OH)_2$

References

[1] Dolomanov, O. V.; Bourhis, L. J.; Gildea, R. J.; Howard, J. A. K.; Puschmann, H. OLEX2: *J. Appl. Cryst.* **2009**, *42* (2), 339–341.

[2] Sheldrick, G. M. *Acta Cryst.* **2015**, *71* (1), 3–8.

[3] Turner, M. J.; McKinnon, J. J.; Jayatilaka, D.; Spackman, M. A. *CrystEngComm* **2011**, *13* (6), 1804–1813.

[4] Wang, J.; Li, X.; Wang, M.; Zhang, T.; Chai, X.; Lu, J.; Wang, T.; Zhao, Y.; Ma, D., *ACS Catal.* **2022,** *12*, (11), 6722-6728.

[5] Liu, F.; Gao, X.; Shi, R.; Tse, E. C. M.; Chen, Y.,. *Green Chem.* **2022,** *24*, (17), 6571-6577.

[6] Zhou, H.; Ren, Y.; Li, Z.; Xu, M.; Wang, Y.; Ge, R.; Kong, X.; Zheng, L.; Duan, H., *Nat. Commun.* **2021,** *12*, (1), 4679.

[7] Liu, X.; Fang, Z.; Xiong, D.; Gong, S.; Niu, Y.; Chen, W.; Chen, Z., *Nano Res.* **2022,** *16*, (4), 4625-4633.

[8] Ma, F.; Wang, S.; Gong, X.; Liu, X.; Wang, Z.; Wang, P.; Liu, Y.; Cheng, H.; Dai, Y.; Zheng, Z.; Huang, B., *Appl. Catal. B. Environ.* **2022,** *307*.

[9] Wang, J.; Li, X.; Zhang, T.; Chen, Y.; Wang, T.; Zhao, Y., *J. Phys. Chem. Lett.* **2022,** *13*, (2), 622-627.

[10] Li, Y.; Zhao, Y.; Zhao, H.; Wang, Z.; Li, H.; Gao, P., *J. Mater. Chem. A* **2022,** *10*, (38), 20446-20452.

[11] Wang, N.; Li, X.; Hu, M.-K.; Wei, W.; Zhou, S.-H.; Wu, X.-T.; Zhu, Q.-L.,. *Catal. B Environ.* **2022,** *316*.

[12] Bashir, I.; McGettrick, J. D.; Kühnel, M. F.; Sarfraz, B.; Arshad, S. N.; Rauf, A.*ACS Sustainable Chem Eng* **2024**, *12* (12), 4795–4802.

[13] Li, J., Li, L., Ma, X., Han, X., Xing, C., Qi, X., He, R., Jordi Arbiol, Pan, H., Zhao, J., Deng, J., Zhang, Y., Yang, Y. and Cabot, A. (2023). *Adv. Sci.*,**2023**, 10(15),2300841.

[14] Jiao, L., Wei, W., Li, X., Hong, C.-B., Han, S.-G., Muhammad Imran Khan and Zhu, Q.-L. **2022**, *Rare metals*, 41(11), pp.3654–3661.
